# Morphophenotypic classification of tumor organoids as an indicator of drug exposure and penetration potential

**DOI:** 10.1371/journal.pcbi.1007214

**Published:** 2019-07-16

**Authors:** Aleksandra Karolak, Sharan Poonja, Katarzyna A. Rejniak

**Affiliations:** 1 Integrated Mathematical Oncology Department, H. Lee Moffitt Cancer Center & Research Institute, Tampa, FL, United States of America; 2 Department of Oncologic Sciences, Morsani College of Medicine, University of South Florida, Tampa, FL, United States of America; University of Virginia, UNITED STATES

## Abstract

The dynamics of tumor progression is driven by multiple factors, which can be exogenous to the tumor (microenvironment) or intrinsic (genetic, epigenetic or due to intercellular interactions). While tumor heterogeneity has been extensively studied on the level of cell genetic profiles or cellular composition, tumor morphological diversity has not been given as much attention. The limited analysis of tumor morphophenotypes may be attributed to the lack of accurate models, both experimental and computational, capable of capturing changes in tumor morphology with fine levels of spatial detail. Using a three-dimensional, agent-based, lattice-free computational model, we generated a library of multicellular tumor organoids, the experimental analogues of in vivo tumors. By varying three biologically relevant parameters—cell radius, cell division age and cell sensitivity to contact inhibition, we showed that tumor organoids with similar growth dynamics can express distinct morphologies and possess diverse cellular compositions. Taking advantage of the high-resolution of computational modeling, we applied the quantitative measures of compactness and accessible surface area, concepts that originated from the structural biology of proteins. Based on these analyses, we demonstrated that tumor organoids with similar sizes may differ in features associated with drug effectiveness, such as potential exposure to the drug or the extent of drug penetration. Both these characteristics might lead to major differences in tumor organoid’s response to therapy. This indicates that therapeutic protocols should not be based solely on tumor size, but take into account additional tumor features, such as their morphology or cellular packing density.

## Author summary

Primary tumors and tumor metastases grow as three-dimensional (3D) masses of cells. Depending on the surrounding stroma, they may acquire various shapes, more or less irregular. Tumor organoids are the 3D experimental cultures that mimic growth of in vivo tumors, as well as their response to treatments. However, it is difficult to assess experimentally in a reproducible and quantitative way, how tumor morphology influences treatment efficacy. Here, we used mathematical modeling and computer simulations to analyze the structure of the simulated organoids and to classify them with regards to two quantitative features: the tumor accessible surface area (ASA) describing organoid exposure to the drug and the extent of drug penetration through the tumor tissue (organoid compactness). We showed that organoids of similar sizes and growth dynamics can, in fact, be characterized by distinct values of compactness and ASA, and thus may respond differently to the drug treatment. We suggest that these tumor features should be taken into consideration in addition to tumor size, when the therapeutic interventions are designed.

## Introduction

Organoid cultures are the three-dimensional (3D) in vitro experimental systems in which individual cells grow and self-organize into multicellular structures that recapitulate the morphology and, to some extent, the functionality of the organ from which they are derived [1, 2]. The complexity of organoid structure rises from single-layered hollow spherical breast or prostate acini [3, 4] to the branched breast or salivary ducts [5, 6] to the multilayered colon polyps [7] or brain lobules [8]. These in vitro cultures are utilized to address a wide range of biological questions, such as testing the mechanisms of tissue development and homeostatic maintenance and the initiation of malignant transformations and cancer growth dynamics, as well as anti-cancer treatments [9–11]. The Nature Publication Group recognized the novelty and importance of the organoid culture system by naming the organoid experiments as the Method of the Year in 2017. Tumor organoids can be derived either from tumor cell lines or a patient’s tumor cells. They are used as tumor surrogates, as they resemble the biological and biophysical features of tumors and metastases better than 2D cell cultures. Also, these in vitro cultures can be controlled more efficiently than mouse experiments. Various tumor types can be currently cultured as organoids, including pancreatic [12], liver [13], gastrointestinal [14], prostate [15], brain [16] or breast [17] tumors. Tumor organoids can acquire diverse morphologies even if they are derived from the same organ. Kenny et al. showed that tumor organoids derived from twenty-five different breast cancer cell lines developed into four different multicellular shapes (round, mass, grape-like and stellate) after four days in culture [18]. Harma et al. obtained similar results for organoids derived from prostate cancer cell lines [19]. This morphological diversity may arise due to intrinsic differences, including distinct genetic profiles [20, 21], or extrinsic factors, such as various oxygenation levels or the extracellular matrix composition [22, 23].

Tumor organoids have also been a subject to mathematical modeling. Various modeling frameworks were employed to investigate organoid development, metabolic variability and interactions with other components of tumor microenvironment. These include cellular automata models [24–27], particle-force models [28–30], Cellular Potts models [31–33], continuous models [34] and our own immersed boundary model [35, 36]. Our recent review [37] provides more details on these models and summarizes latest achievements in the mathematical modeling of tumor organoids. The model presented here was first developed in [38] and belongs to the class of particle-force models. The novelty of our studies lies in considering quantitatively a range of biologically relevant features to simulate a library of heterogeneous tumor organoids, and in developing new methods to quantify organoids’ properties and their potential response to drugs.

There is a growing interest in using tumor organoids for drug screening in order to design more effective treatment combinations and schedules [39–41]. In a typical drug screening experiment, the drug is dissolved in a medium that surrounds the growing organoid and drug effectiveness is evaluated based on the changes in organoid size that are recorded over time. Usually, the bright field images of tumor organoid cross sections are used to determine two diameter measurements, the longest distance between opposite sides of the tumor (length, L) and the distance between tumor sides taken along the perpendicular direction (width, W). Subsequently, these values are used to report either the average tumor diameter (W+L)/2, tumor area W*L, or tumor volume (L*W*W)/2 [42]. These measurements reflect well the changes in the tumor mass if the organoids are of spherical shape. However, since organoid morphologies are often irregular and deviate from a sphere, this approach can lead to an inaccurate assessment of the organoid’s area or volume and, consequently, an incorrect measure of the organoid’s response to treatments. To avoid this discrepancy, a perimeter was proposed to better approximate the organoid’s volume from the 2D images [43]. These different morphologic measurements (diameter, perimeter, area or volume) were incorporated into the automatic analyses and classifications of tumor morphologies [44, 45]. However, despite the technical and computational advances engaged in comparing tumor morphological features and their effects on drug exposure, many aspects related to tumor compactness and circularity remain elusive. Therefore, novel methods for assessing how tumor morphophenotypic properties impact drug dose estimation and therapy responses, which depart from evaluations based on organoid’s diameter, would provide a valuable independent prognostic factor.

In this study, we generated a large number of in silico organoids, all with similar growth dynamics that fit the same set of tumor diameter measurements. In order to avoid effects of nutrient diffusivity limits, we restricted our simulations only to the initial phase of organoid development when the simulated structures are less that 150 microns in radius. Larger organoids may develop internal regions of hypoxia and necrosis that could influence the efficacy of administered treatments. We showed that the organoids we simulated attained morphologies, which can be classified into four groups (morphophenotypes) that describe organoids exposure to and penetration by the drug and, consequently, their potential response to the treatment. Since assessments of tumor diameter are often under- or overestimated, our classification methods are diameter-free but take advantage of organoid compactness and circularity measurements. The in silico organoids were simulated using the 3D framework, *MultiCell-LF* (the Multi-Cellular Lattice Free framework), by varying three biologically-relevant parameters: cell age at division (*A*_*div*_), cell contact inhibition controlled by the number of surrounding neighbor cells (*N*_*neigh*_) and the maximal cell radius (*R*_*max*_). The considered ranges of these parameters cover the biological features of distinct cell types [20, 46, 47] grown in various microenvironments [19, 20, 22, 46, 47]. The library of organoid morphologies generated from the sampled parameter space was categorized into morphophenotypic classes, and the impact of each parameter on tumor morphology was evaluated. Additionally, we used two metrics originating from the proteins’ structural biology, i.e., the radius of gyration (*RGYR*) and the accessible surface area (*ASA*), [48–51], as the principal classification criteria. They together provided comprehensive and diameter-free analysis that may help identify the presence of patches, buried niches and extended chains, as well as compact or loose tumor masses that may change how receptive to the treatment the organoid is. The idea behind this approach is to use mathematical modeling and computer simulations to delineate the basic principles of global and local features of tumor organoid organization and, to quantify irregularities in organoid morphology. Our long-term goals are to apply this in silico screening approach to the experimental tumor organoids grown in diverse microenvironmental conditions and to test hypotheses suggesting improvements in cancer treatment. Here, we present the first step in this direction by applying our classification criteria to the computer-generated organoid morphologies.

## Methods

### Computational *MultiCell-LF* model

#### Individual cells

The off-lattice, agent-based model is used to define individual cells and their physical interactions. Each cell *C*_*i*_ is represented in the 3D space by its centroid (cell nucleus) with coordinates ***X***_*i*_ and a current cell radius *R*_*i*_ that changes during cell growth from 0.65*R*_*max*_ at its birth to the maximal value *R*_*max*_ when the cell is mature. Each cell lifespan is traced with an individually regulated cell cycle, the current cell age *A*_*i*_ and the cell division age *A*_*i*,*div*_ at which the cell is ready to divide. However, the cell proliferation process can be halted due to contact inhibition that is defined by the number of the cell’s immediate neighbors *N*_*i*_. If *N*_*i*_ exceeds the prescribed number *N*_*neigh*_, the cell remains quiescent until this condition changes. Cells interact physically with their neighbors via repulsive-adhesive forces. Repulsive forces ensure that each cell’s volume is preserved by pushing the neighboring cells apart if they come too close. Adhesive forces allow for the formation of compact, multicellular organoids by providing physical links between individual cells. All cell-cell interactions are defined locally between individual cells, and the overall tumor growth dynamics and morphology are the emergent properties of the collective actions of many individual cells.

#### Cell-cell repulsions

Each cell has a volume that is maintained during its lifetime. If the neighboring cells *C*_*i*_ and *C*_*j*_ come into contact, i.e., if the distance between the cells’ nuclei is less than the sum of the cells’ current radii *R*_*i*_ + *R*_*j*_, they push each other away by exerting repulsive forces until their volumes are restored. These forces are modeled as linear Hookean springs. Thus, the repulsive force exerted on cell *C*_*i*_ is defined as

$$f_{i,j}^{rep}=\left\{ \begin{array}{l} F^{rep}\;((R_{i}+R_{j})-\|X_{i}-X_{j}\|)\frac{X_{i}-X_{j}}{\|X_{i}-X_{j}\|}\;if\;\|X_{i}-X_{j}\|<R_{i}+R_{j}\\ \;0\;otherwise, \end{array}\right.$$ (1)

where *F*^*rep*^ is the constant spring stiffness and the spring resting length is equal to the sum of the cells’ current radii *R*_*i*_ + *R*_*j*_. If the cell *C*_*i*_ is in the neighborhood of several cells *C*_*j1*_, …, *C*_*jM*_, the total repulsive force **F**_*i*_^*rep*^ acting on *C*_*i*_ is the sum of the repulsive forces coming from each neighboring cell. Thus, the total repulsive force is given by ***F***_*i*_^*rep*^ = ***f***_*i*,*j1*_^*rep*^ +…+***f***_*i*,*jM*_^*rep*^.

#### Cell-cell adhesions

If two cells *C*_i_ and *C*_*j*_ in the organoid are pushed apart further than the maximal cell diameter 2*R*_*max*_, the adherent forces are activated to ensure organoid compactness. However, only cells located within the distance smaller than 2.25*R*_*max*_ are taken into consideration to avoid activation of adhesive forces between cells that are located too far away. In this case, the Hookean force exerted on cell *C*_*i*_ is given by:

$$f_{i,j}^{adh}=\left\{ \begin{array}{l} F^{adh}\;(2R_{max}-\|X_{i}-X_{j}\|)\frac{X_{i}-X_{j}}{\|X_{i}-X_{j}\|}\;if\;2R_{max}<\|X_{i}-X_{j}\|<{2.25R}_{max}\\ \;0\;otherwise, \end{array}\right.$$ (2)

where *F*^*adh*^ is the constant spring stiffness and 2*R*_*max*_ is the spring resting length. As before, if *M* neighboring cells exerts adhesive forces, the total adhesive force **F**_*i*_^*adh*^ acting on *C*_*i*_ is the sum of the adhesive forces coming from each cell in the *C*_*i*_ neighborhood, i.e., ***F***_*i*_^*adh*^ = ***f***_*i*,*j1*_^*adh*^ +…+***f***_*i*,*jM*_^*adh*^.

#### Cell passive relocation

The total force ***F***_*i*_ exerted on the cell *C*_*i*_ is a sum of all forces (adhesive and repulsive) between that cell and its neighbors. As a result, *C*_*i*_ is passively moved away from its current location. Cell motion is governed by the overdamped spring in which each cell returns to equilibrium without oscillations. The damping force is related linearly to cell velocity with a damping coefficient *η*:

$$F_{i}=\eta \frac{dX_{i}}{dt},\mathrm{where}\;F_{i}=F_{i}^{adh}+F_{i}^{rep},$$ (3)

and, after discretization with a time step *Δt*, the cell’s new position is given by:

$$X_{i}(t+\Delta t)=X_{i}(t)+\Delta tF_{i}/\eta .$$ (4)

#### Cell cycle

Each cells’ progression through the cell cycle is traced separately. The cell-specific division time *A*_*i*,*div*_ is split into four phases that correspond in length to the phases of the mammalian cell cycle [52, 53]. Cell growth takes place predominantly in the interval gap 1 phase (G1) that lasts for 45% of the cell’s lifespan; the synthesis phase (S) corresponds to the time needed for DNA replication and takes 35% of the cell’s lifespan; during the gap 2 phase (G2), the cell can still grow until it reaches the predefined size (duration of 15% of the cell lifespan); finally, the cell divides and produces two daughter cells during the mitosis (M) phase (5% of the cell’s lifespan). Cells may become arrested in their cell cycle due to contact inhibition by neighboring cells. In such cases, they remain metabolically active but do not proliferate [54]. The time of cell arrest does not count toward the length of individual cell cycle phases, and when the halting condition changes, the cell resumes its cell cycle. A different color represents each cell cycle phase in the figures (G1: light yellow, S: dark yellow, G2: orange; M: brown; arrested cells: red).

#### Cell division

Each cell *C*_*i*_ is permitted to divide if its current age *A*_*i*_ reaches the cell division age *A*_*i*,*div*_, and the cell is not a subject to contact inhibition. Upon the division of the cell *C*_*i*_, the two daughter cells *C*_*i1*_ and *C*_*i2*_ are created instantaneously. The daughter cells are placed symmetrically around the nucleus of the mother cell within a distance of 0.5*R*_*max*_:

$$\begin{array}{c} X_{i1}=X_{i}+0.5R_{max}(\sin\;\theta \;\cos\;\varphi ,\sin\theta \;\sin\varphi ,\cos\;\theta ),\\ X_{i2}=X_{i}-0.5R_{max}(\sin\theta \;\cos\;\varphi ,\sin\theta \;\sin\varphi ,\cos\;\theta ), \end{array}$$ (5)

where the azimuthal angle *θ* is chosen randomly within the interval [0, *π*], the equatorial angle *ϕ* is chosen randomly from [0,2*π*), and *R*_*max*_ is the maximal cell radius. The current age of each daughter cell is initialized to zero. The division age for each daughter cell (*A*_*i1*,*div*_ and *A*_*i2*,*div*_) is equal to the base value of *A*_*div*_ with a small noise term to avoid synchronized division of a large cell subpopulation. The current radius of each cell is set up to 0.65*R*_*max*_. Since both daughter cells are placed at a distance smaller than their current diameters, the repulsive forces between them are activated. Both daughter cells may also experience multiple repulsive and adhesive forces from other neighboring cells, which will be applied to push the cell until the whole tumor cell cluster reaches an equilibrium configuration.

#### Cell contact inhibition

In off-lattice agent-based models, each cell may have a different number of immediate neighbors. The contact inhibition criterion is evaluated by inspecting how many cells are located within a prescribed distance from the host cell. The cell is subjected to contact inhibition if the number of neighboring cells *N*_*i*_ located within two cell diameters exceeds the predefined number of neighbors *N*_*neigh*_.

#### Initial conditions

Each simulation starts from a single cell with a defined cell size, cell division age and sensitivity to contact inhibition. The cell progresses through its cell cycle and gives rise to two daughter cells that inherit all properties of the mother cell except for the division age, which is perturbed by a small random noise term to avoid the synchronization of the whole cluster of cells. As this process continues, each cell that is not contact-inhibited will divide upon reaching its division age. All simulated results are reported after 14 days of the simulated time.

### Organoid morphological features

#### Calculations of the diameter of a simulated organoid

The diameter (*D*) of a simulated organoid is an average of three diameter measurements from 2D projections onto the *xy*, *yz*, or *xz* planes.

#### Correlation between simulated and test data

The test data represents values of organoid’ diameters at five time points (days 0, 3, 7, 10 and 14). We followed the trend from [20] that reported sizes of two malignant cell lines with different metastatic capabilities grown in 3D cultures. The morphologies of these organoids were reported in [55] and used as a benchmark for our simulations. The average values were used to determine an optimal fitting curve and the simulated and test data were compared using the correlation criterion of *R*^2^ >0.9, where *R*^*2*^ is the statistical coefficient of determination. Only the simulations that satisfied this criterion were used for further morphological analysis.

#### Radius of gyration

The radius of gyration (*RGYR*) refers to the distribution of an object’s components around its center of mass. This term is commonly used in structural biology to determine the compaction of a molecule. The larger the *RGYR* value the lower the compaction. For example, the *RGYR* value for *α* helix proteins, which have the least compact secondary structure, is the largest, with *β* and α +*β* proteins following [51]. Here, *RGYR* is employed to determine the compactness of each organoid:

$$RGYR=\sqrt{\sum_{i=1}^{N}{\left\|X_{i}-X_{C}\right\|}^{2}/N_{cells}},$$ (6)

where *X*_*i*_ is the center of the *i*^th^ cell, *X*_*C*_ is the center of mass of the organoid, and *N*_*cells*_ is a number of cells forming the organoid.

#### Organoid accessible surface area

Since the organoid is a conglomerate of individual cells, its surface is composed of several convex spherical caps that adhere to one another. Each of these sub-surfaces is accessible to the drug and contributes to the overall drug absorption. Thus, the amount of drug that is taken in by the whole organoid (either via membrane diffusion or receptor binding) depends on the surface area accessible to the drug. We borrowed the concept of the accessible surface area (*ASA*) from molecular biology, where it is used to denote a surface area of an irregular 3D biomolecule that may be in direct contact with an external solvent. Here, we use this concept as a measure of the exposure of the tumor organoid surface to the external medium. Since the overall organoid surface is not smooth, it can only be calculated numerically. We followed implementation from the Visual Molecular Dynamics software (VMD) [49]. In this algorithm, 500 points are randomly distributed around each cell at a distance larger than the cell radius to represent a 1/500^th^ portion of the surface area of that cell. If the points belonging to one cell fall within the volume of another cell, they are removed from calculations to account for contact between neighboring cells. In this way all points located inside the organoid are removed, since the intercellular spaces do not contribute to the external surface area of the organoid. The surface areas of each spherical cap corresponding to the points that were not removed are added up to count for the final *ASA* value.

## Results

### Exploration of the *MultiCell-LF* parameter space

**Fig 1:**
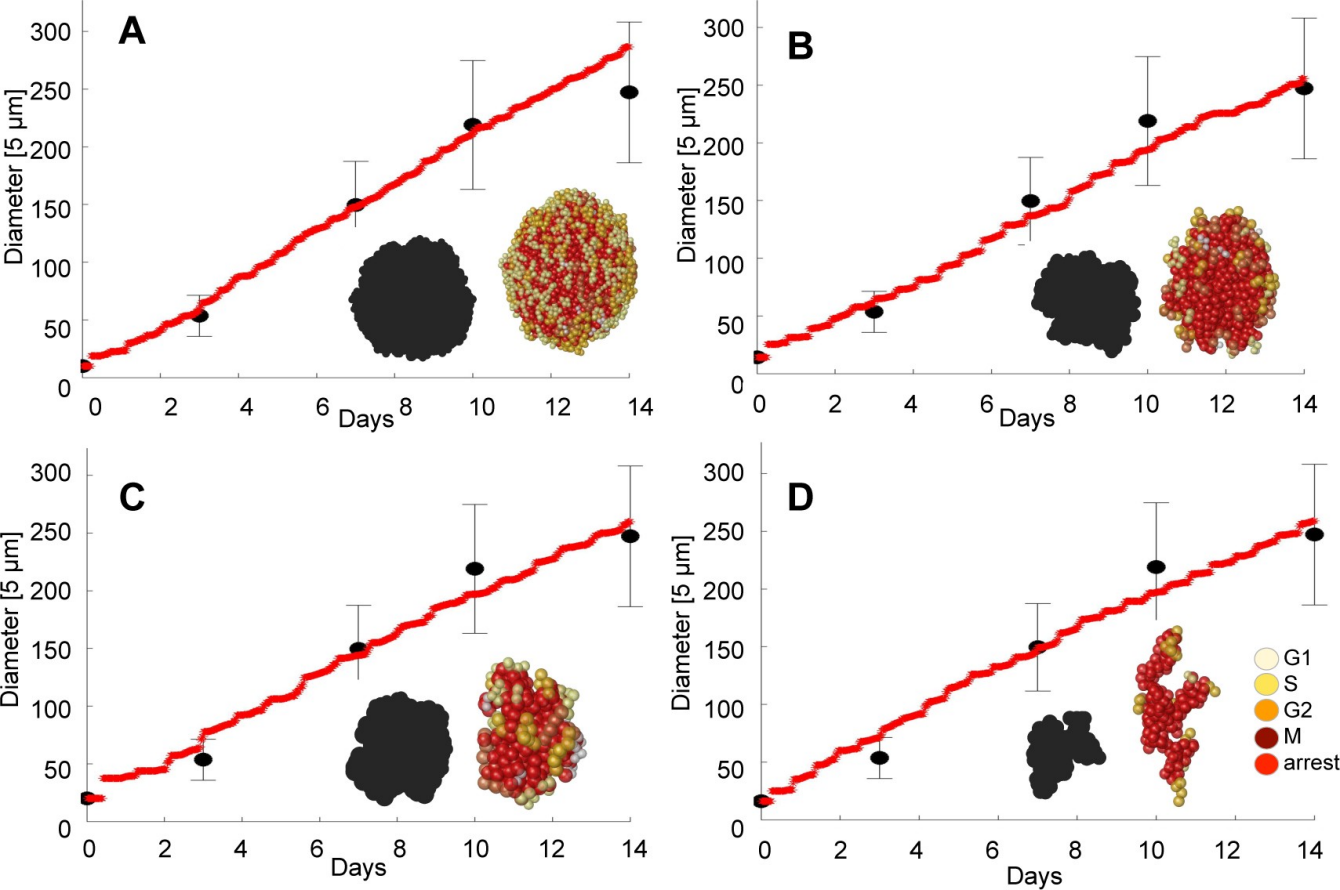
Representative simulated data. **A-D.** Four simulated organoids reproducing the test data with correlation *R*^2^>0.9. Red lines show simulated organoid diameters. Black dots represent averaged diameters of the test data at days 0, 3, 7, 10 and 14, vertical lines show standard deviations. Insets show final organoid morphologies and their projections on the xy plane. Simulated cases: **A.** *R*_*max*_ = 5 μm, *A*_*div*_ = 11 hours, *N*_*neigh*_ = 15 cells, *R*^2^ = 0.987; **B.** *R*_*max*_ = 7 μm, *A*_*div*_ = 13 hours, *N*_*neigh*_ = 9 cells, *R*^2^ = 0.989; **C.** *R*_*max*_ = 10 μm, *A*_*div*_ = 20 hours, *N*_*neigh*_ = 11 cells, *R*^2^ = 0.986; **D.** *R*_*max*_ = 8 μm, *A*_*div*_ = 13 hours, *N*_*neigh*_ = 6 cells, *R*^2^ = 0.983. Cell colors represent the cell cycle phases: G1: light yellow, S: dark yellow, G2: orange; M: brown; the cells arrested during the cell cycle: red.

Our first goal was to examine the diversity of the simulated organoids under the constraint that their diameter measurements fit the test data. We explored three cellular features, which could affect the generated organoid morphology: the radius of a tumor cell *R*_*max*_; the cell doubling time defined as the age at which the cell is ready to divide *A*_*div*_; and the cell’s sensitivity to contact inhibition, defined as the number of cell neighbors *N*_*neigh*_ that will halt the cell proliferation process. Three sets of simulations were performed for each parameter selection, and only those cases were further analyzed for which all three simulations fitted the test data. In each case, we only used the diameter information of the simulated organoids for comparison to the test data. Only those simulations that fulfilled both criteria: (i) were within the average +/- std diameter values of the test set, and (ii) the correlation between the diameters of the simulated organoids and the test data satisfied *R*^2^>0.9, were accepted for further analysis. Four representative examples characterized by different *R*_*max*_, *A*_*div*_ and *N*_*neigh*_ values are shown in Fig 1A–1D.

**Fig 2:**
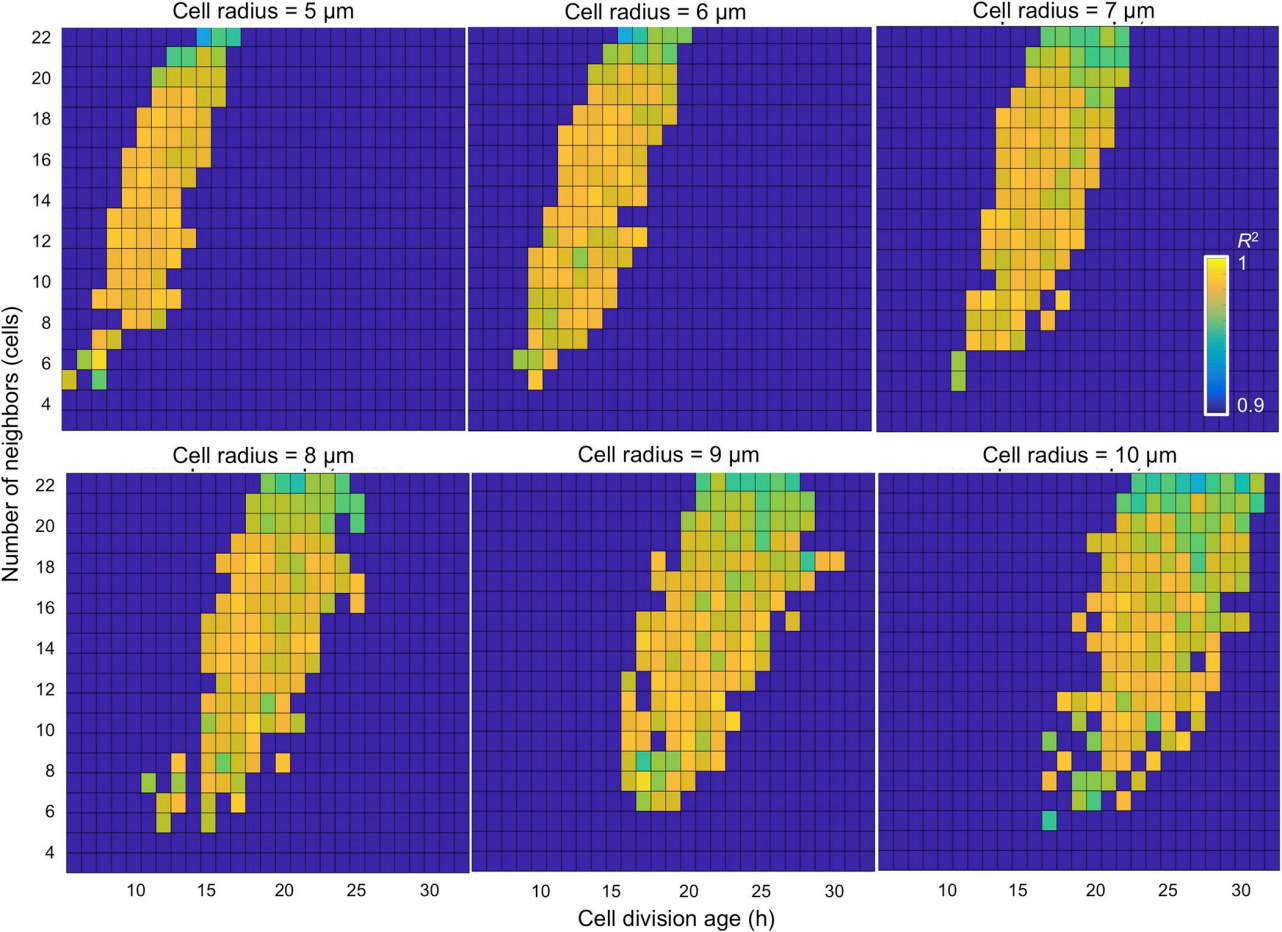
Heatmap analysis of the correlation between simulated and test data. Heatmaps of the sampled parameter space for six different values of cell radius *R*_*max*_ (5 to 10 μm), cell division times *A*_*div*_ (between 6 and 32 hours), and the number of cell neighbors *N*_*neigh*_ (between 3 and 22 cells). Shown are only the cases with *R*^2^ >0.9.

In our analysis, we considered a wide range of model parameters. *R*_*max*_ was varied between 5 μm and 10 μm, and these values are consistent with cell sizes reported in the literature [56, 57]. *A*_*div*_ was varied between 6 and 32 hours that has also been observed in experiments for different tumor cell lines [57, 58]. *N*_*neigh*_ was varied between 3 and 22 cells to mimic cell sensitivity to contact inhibition that is directly related to the density of neighboring cells. The resulting heatmaps of the sampled parameter space organized by increasing cell radius are shown in the panels of Fig 2. The generated parameter space covered an area of heterogeneous phenotypic and genotypic features. For example, different cell sizes (values of *R*_*max*_) may correspond to cells of a different origin or from distinct cell lines. A cell’s sensitivity to contact inhibition (controlled by *N*_*neigh*_) may be representative of tumor cell invasive properties from epithelial-like to mesenchymal cells. The cell doubling time (defined by *A*_*div*_) may be associated with a specific tumor type or tumor cell line, and may correspond to a cell’s aggressiveness level or the microenvironmental condition (acidity, oxygenation, nutrient contents) in which the cells are growing.

### Model morphochart space

**Fig 3:**
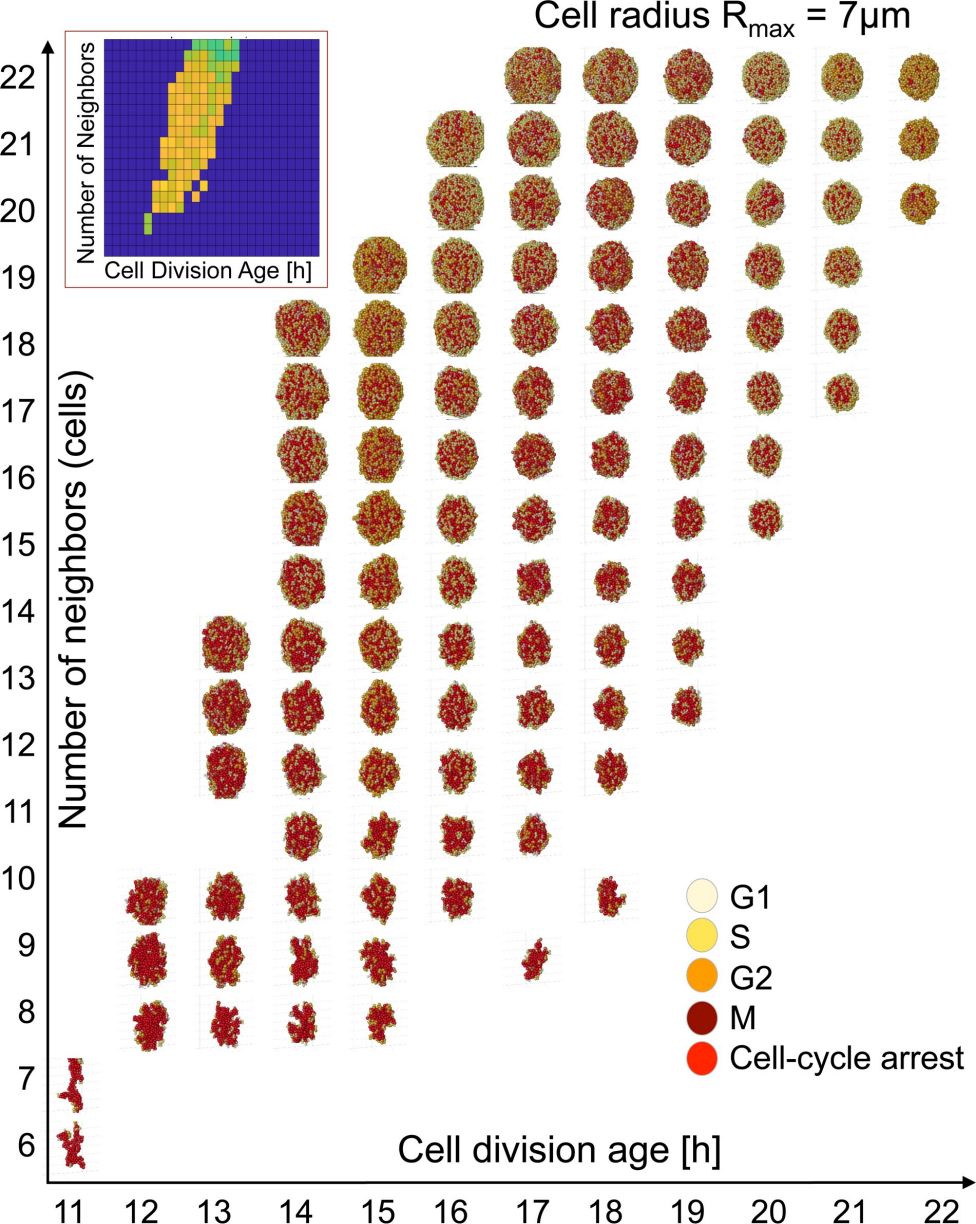
3D Organoid morphochart. A collection of final morphologies (a morphochart) of simulated organoids with a fixed cell radius of *R*_*max*_ = 7 μm, cell division *A*_*div*_ varied between 11 and 22 hours, and cell neighbor number *N*_*neigh*_ varied between 6 and 23 cells. Only results with *R*^2^ >0.9 are shown. The inset shows a corresponding heat map from Fig 2. Cells are colored according to their phase of the cell cycle, as described in Fig 1.

The heatmaps in Fig 2 provided an evaluation of how well the diameter measured during in silico organoid growth correlated with the test data. In addition to diameter measurement, our model also generated the organoid morphocharts, which are collections of the final organoid morphologies. The representative morphochart for organoids with a fixed cell radius of *R*_*max*_ = 7 μm shown in Fig 3 revealed that, within constrains of our correlation criterion, a broad spectrum of organoid morphologies arose when the three cellular parameters selected for our study were varied.

**Fig 4:**
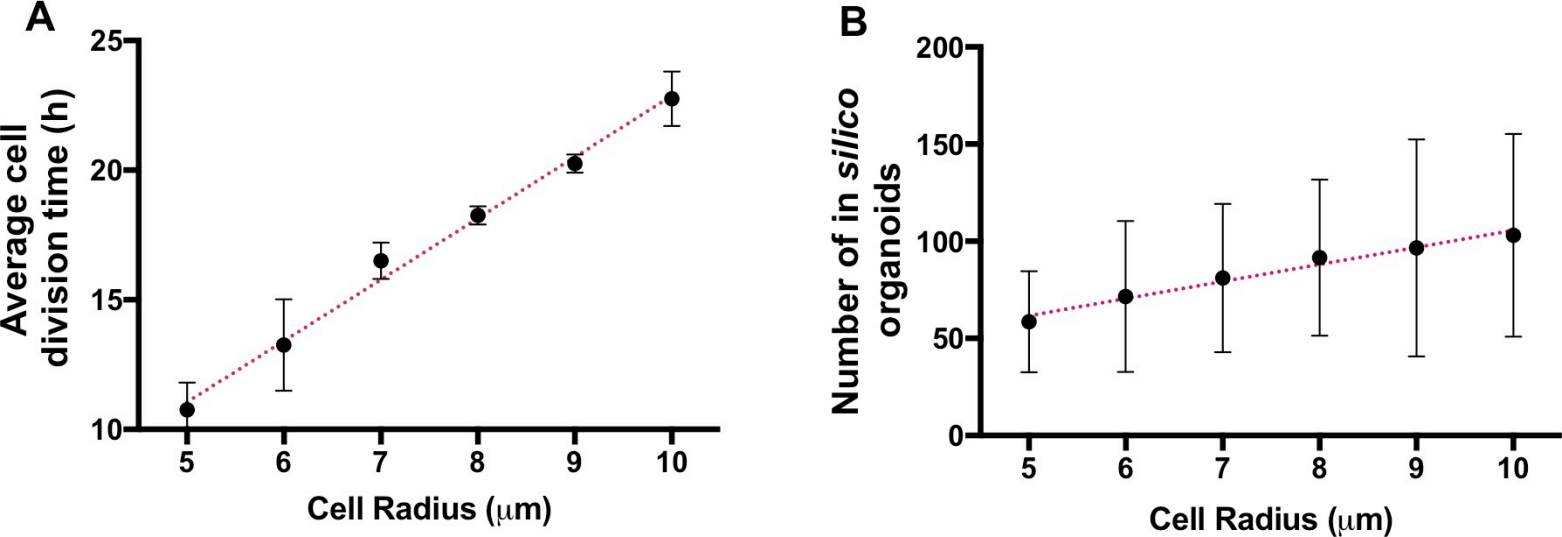
Quantitative analyses of organoids’ heatmaps. **A.** An average cell division age *A*_*div*_ as a function of *R*_*max*_. **B.** An average number of simulated organoids that fulfill the correlation criterion as a function of *R*_*max*_. The error bars represent std from three independent simulations for a given set of parameters.

Close inspection of this radius-specific morphochart shows that the organoids tend to attain irregular shapes for low values of *N*_*neigh*_ (strict contact inhibition). This effect is more profound for rapidly dividing cells (small value of *A*_*div*_). When the values of *N*_*neigh*_ increase (reduced contact inhibition) the organoids’ shapes become more regular and reach morphology close to an ideal sphere for the highest values of *N*_*neigh*_. The *N*_*neigh*_ also has a robust effect on the final organoid size. The organoids grow smaller (within the average +/- std values) when *N*_*neigh*_ is reduced compared with those having high *N*_*neigh*_. This relationship between *N*_*neigh*_ and tumor shape is preserved in all morphocharts (S1–S6 Figs). Additionally, the organoid’s size and shape depend on *A*_*div*_, and, for each row with fixed *N*_*neigh*_, the size of the simulated organoids decreases with an increasing *A*_*div*_. Contrasting the contact inhibition parameter, the range of *A*_*div*_ for which the simulated organoids fit the experimental data (as observed in the heatmaps in Fig 2) shows a strong dependency on the cell radius. For example, for *R*_*max*_ = 5 μm, the parameter *A*_*div*_ can be varied between 6 and 17 hours, while *A*_*div*_ spans from 17 to 32 hours for *R*_*max*_ = 10 μm (the range of *A*_*div*_ values for each cell radius is shown in Fig 4A). This confirms that larger cells must be characterized by a slower division rate to fit the experimental organoid dynamics of growth.

Interestingly, not only is the range of *A*_*div*_ different for cells of different sizes, but the total number of simulated organoids that fulfill the correlation criteria also increases with a larger cell size; i.e., the parameter space consists, on average, of about 50 organoids for *R*_*max*_ = 5 μm and 100 for *R*_*max*_ = 10 μm (Fig 4B). Since the organoids simulated for each value of *R*_*max*_ are fitted and correlated to the same growth dynamics data, it seems that cells characterized by small radii are less likely to adapt to changes in their vicinity and only a narrow valley in model parameter space fits the behavior of in vitro organoids. These parameter combinations are limited by small cell size, which demands faster division times *A*_*div*_. At the same time, the simultaneous fluctuations of *A*_*div*_ and *N*_*neigh*_ provide higher flexibility, allowing the simulated organoids with larger cells to fulfill the correlation criteria.

### Organoid structural quantification

Since the generated organoids attain different morphologies, we examined whether they possess any common structural characteristics. We analyzed the compactness and accessible surface area across all in silico organoids. These two metrics were chosen to assess how effectively the externally supplied chemotherapeutic agent would reach all the organoid cells. Therefore, we tested what portion of the given organoid was directly exposed to the externally supplied drug (organoid accessible surface area), and how easy it would be for the drug to penetrate the organoid (organoid compactness).

**Fig 5:**
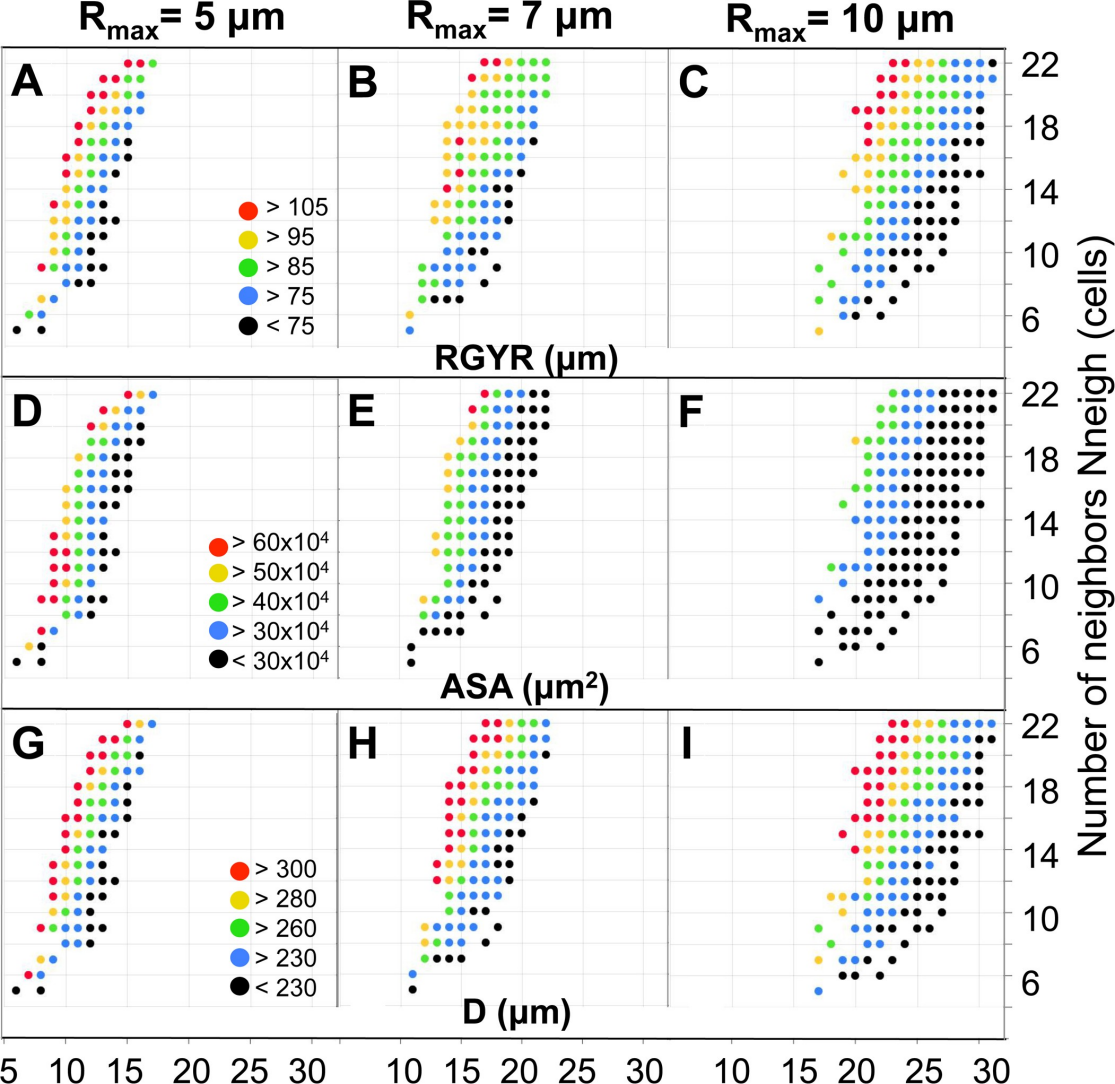
Organoids’ structural properties. Color-coded classification of: **A-C.** Radius of gyration: *RGYR*. **D-F.** Accessible surface area: *ASA*. **G-I.** Organoid diameter: *D*, for organoids with cells of radii *R*_*max*_ = 5, 7 and 10 μm, respectively.

To assess organoid compactness, we calculated the organoid’s radius of gyration (*RGYR*), which quantifies the distribution of all cells around the organoid’s center of mass. The larger the *RGYR* value, the less compact the organoid. The color-coded values of *RGYR* plotted as a function of cell division age (*A*_*div*_) and the number of cell neighbors (*N*_*neigh*_) for all organoids with a cell radius of *R*_*max*_ = 5, 7, and 10 μm are shown in Fig 5A–5C, respectively. In all three cases, the organoid compactness decreased when the cells could divide more often (low *A*_*div*_) or when they were less sensitive to contact inhibition (larger *N*_*neigh*_) to halt their cell cycle progression (red dots). For the same final morphologies, the accessible surface area (*ASA*) was calculated, and three color-coded heatmaps for the corresponding cellular radii are shown in Fig 5D–5F. While each heatmap separately contains a similar pattern of higher *ASA* values for organoids with faster growing cells and larger *N*_*neigh*_, there is a visible shift in the *ASA* values for increasing *R*_*max*_, which contrasts with *RGYR*. This observation indicates that *ASA* is dependent on the cell size. Organoids with cells with a smaller *R*_*max*_ have increased surface area, while larger cells tend to “smooth out” *ASA*. The actual diameter values (*D*) of each generated organoid are shown in Fig 5G–5I. Since there is variability in the sizes of experimental organoids, the generated in silico organoids were accepted if their growth dynamics did not exceed the average +/- std values of the test organoid set. Fig 5G–5I and Fig 5A–5C show good correlation between the patterns of the in silico organoid diameter and *RGYR* values, which implies that organoid’s compactness depends on its size, with less-compact organoids (high *RGYR*) having a larger diameter.

### ASA implies that organoids with similar diameters may require different drug doses

While there is a positive correlation between the values of *D* and *ASA* for the whole organoid library shown in Fig 5, the patterns do not overlap as closely as between *D* and *RGYR*. Instead, there are organoids within the same diameter range (indicated by the same color in Fig 5G–5I) but with distinct *ASA* values (analogous data points have different colors in Fig 5D–5F). For example, organoids represented by red dots in Fig 5H (i.e., with *R*_*max*_ = 7 μm, *N*_*neigh*_ = 21 cells, *A*_*div*_ = 18 hours and *A*_*div*_ = 16 hours) have the highest values of *D*, while their *ASA* values (analogous points in Fig 5E) span a wider range of values: from 30x10^4^ μm^2^ to above 60x10^4^ μm^2^, encompassing a difference of four color bins. Consequently, we observe that significantly different *ASA* values might be found for organoids with comparable sizes and the same cell radius.

This observation directs attention to organoids with comparable diameters but with evident differences in morphology. For example, the organoids represented by blue dots in Fig 5H (i.e., *A*_*div*_ = 17 hours, N_neigh_ = 16 cells; *A*_*div*_ = 13 hours, N_neigh_ = 9 cells; *A*_*div*_ = 11 hours, N_neigh_ = 6 cells; all with *R*_*max*_ = 7 μm) have diameters between 230–260 μm but have significantly different *ASA* values (31.07x10^4^ μm^2^, 41.35x10^4^ μm^2^ and 12.79x10^4^ μm^2^, respectively) and diverse numbers of cells within each organoid (1659, 935, and 204, respectively). These organoids also belong to two different morphophenotypic classes (next section), therefore, the differences in *ASA* calculated for morphologically diverse organoids with similar sizes might provide insights into the importance of correctly estimating the morphological features of the tumor to make proper predictions. Here, under- or overestimation of tumor diameters from 2D images could lead to a highly inaccurate assessment of organoid exposure-related features.

### Radius of gyration and accessible surface area determine distinct organoid morphophenotypes

**Fig 6:**
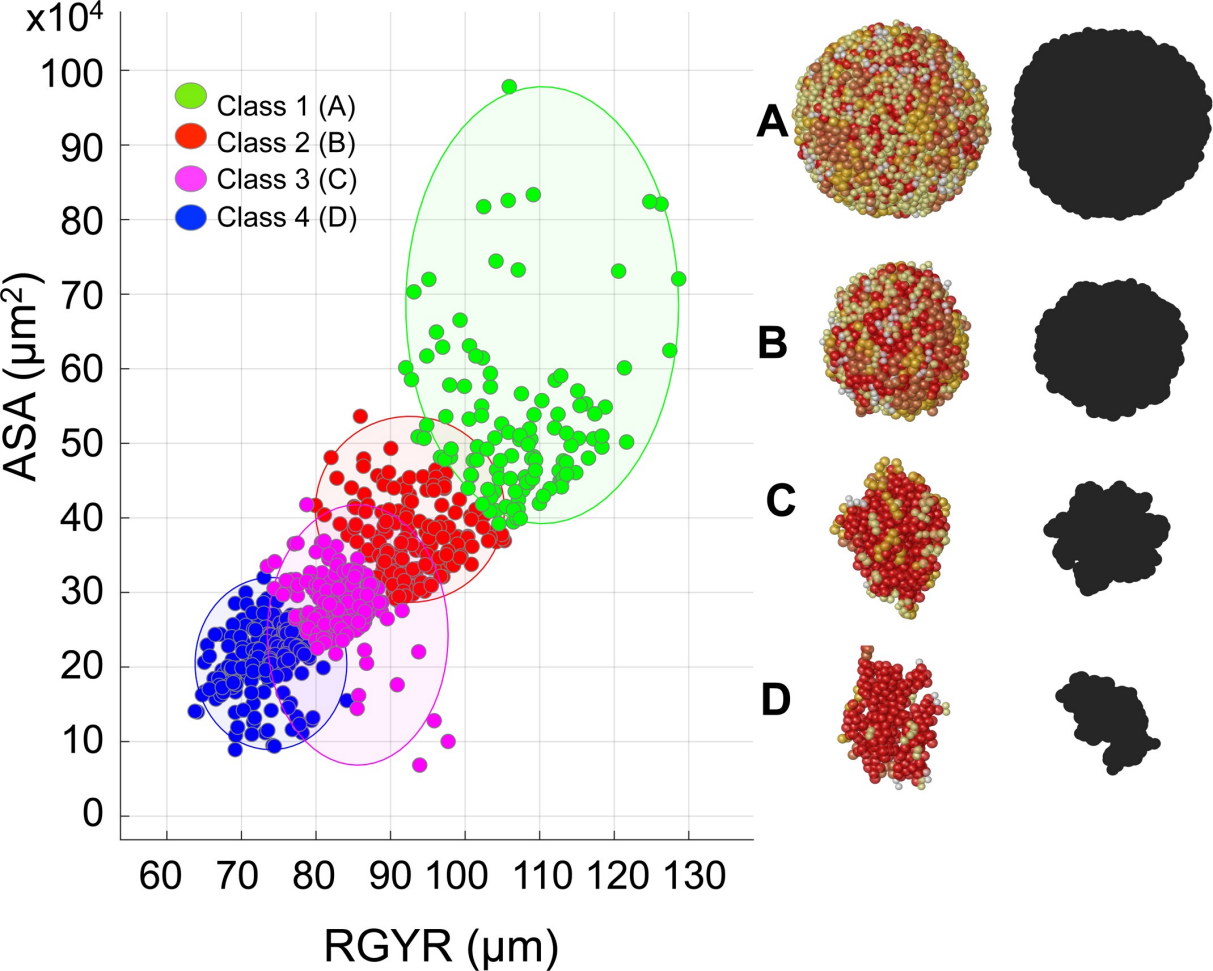
Organoid morphophenotype classification. All simulated organoids divided into four morphophenotypic classes based on their radius of gyration *RGYR* (μm) and accessible surface area *ASA* (x10^4^ μm^2^) using the k-median algorithm: **Class 1**: Spherical; **Class 2**: Compact; **Class 3**: Elongated; and **Class 4**: Branched. The representative organoid morphologies and their xy-plane projections (black shapes) mimicking bright field microscopy images shown: **A** Class 1: *A*_*div*_ = 17 hours, *N*_*neigh*_ = 22 cells, *RGYR* = 127.44 μm, *ASA* = 62.44x10^4^ μm^2^; **B** Class 2: *A*_*div*_ = 18 hours, *N*_*neigh*_ = 19 cells, *RGYR* = 93.32 μm, *ASA* = 32.62x10^4^ μm^2^; **C** Class 3: *A*_*div*_ = 15 hours, *N*_*neigh*_ = 9 cells, *RGYR* = 77.57 μm, *ASA* = 30.97x10^4^ μm^2^; **D** Class 4: *A*_*div*_ = 13 hours, *N*_*neigh*_ = 7 cells, *RGYR* = 73.73 μm, *ASA* = 18.13x10^4^ μm^2^; all four organoids have cell radius *R*_*max*_ = 7 μm.

Joint analysis of the structural and morphological properties of the generated organoids categorized them into four distinct morphophenotypic classes (Fig 6 and Table 1). Class 1 is characterized by high values of both *RGYR* and *ASA*; consequently, the organoids have a large accessible surface area but are not tightly packed. The representative morphology is of a regular spherical shape (Fig 6A). Class 2 also attains quite regular shapes and large values of *ASA*, but smaller values of *RGYR*, resulting in a more compact organoid structure (Fig 6B). In Class 3, the organoid morphologies are elongated (Fig 6C) and are accompanied by smaller *ASA* and relatively small *RGYR* values. Finally, in Class 4, the organoids are characterized by more fragmented, branched shapes (Fig 6D) with small *RGYR* and *ASA* values. This classification was achieved using the classical k-medians clustering algorithm, and the consensus cluster centroids for each class are listed in Table 1.

**Table 1:** Cumulative classification of organoid morphophenotypes.

Class	1	2	3	4
*Cluster Centroid (median)*:				
*RGYR* (μm)	107.33	92.86	82.36	73.70
*ASA* (x10^4^ μm^2^)	50.50	36.42	27.76	21.38
Morphology	Spherical	Compact	Elongated	Branched

These in silico morphophenotypes are similar to the organoid morphologies observed in vitro. Experiments with the organoids derived from 25 different breast tumor cell lines [18] and from 29 different prostate tumor cell lines [19] identified several characteristic organoid shapes, including “Round” spheroids with well differentiated, tightly packed cells similar to our Class 2 “Compact” structures; a “Mass” phenotype characterized by a lower level of cell-cell contacts and cell polarization, resulting in large masses of more loosely packed cells similar to our Class 1 “Spherical” structures; a “Stellate” phenotype characterized by multiple projections and an elongated shape similar to our Class 3 “Elongated” phenotype; and a “Grape-like” phenotype lacking robust cell-cell adhesions and showing a more branched structure similar to our Class 4 “Branched” structures. While there is no complete agreement whether organoid shapes and the level of genetic transformations in the individual cells are correlated, the more aggressive and invasive cell lines tend to have more branched and elongated morphologies and less pronounced cell-cell adhesions, which is also observable in our simulations.

## Discussion

The aberrant morphologies of multicellular in vitro cultures are the first visual indication of cells’ abnormality and potential tumorigenic capabilities. The morphologies of tumor organoids can be quite diverse [18–20, 55] that poses a question whether a given chemotherapeutic treatment will have the same effect on such heterogeneous tumors. Typically, tumor cell response to the drugs is defined by the IC_50_ value (the concentration of an inhibitor where the response is reduced by half) and examined using the 2D monolayer cultures when all cells are well exposed to the therapeutic compounds. However, tumors grow as 3D multicellular conglomerates and thus the differences in individual cell exposure to the drug and the extent of drug penetration though the whole tumor tissue play a paramount role in treatment efficacy. Even the most potent drug will be ineffective if it cannot reach all tumor cells in efficient quantities. This aspect cannot be examined in the 2D cell cultures, thus there is a growing interest in using tumor organoids for testing anti-cancer treatments. However, current experimental protocols do not take into consideration morphological and structural diversities between tumor organoids. Here, our goal was to draw attention to these overlooked aspects of anti-cancer treatment testing. We used a theoretical approach to evaluate the impact of cells’ intrinsic properties and cell-cell interactions on the emergent tumor shape. To the best of our knowledge, this is the first comprehensive computational study that systematically and quantitatively explores morphological diversity of 3D tumor organoids.

The minimalistic set of cell properties considered here included cell size, division time and sensitivity to contact inhibition, all of which can vary from one cell line to another, and between different tumor types. The exploration of these three model parameters gave rise to a library of organoids with diverse morphologies. However, for all these organoids, their average diameters measured during each simulation matched the pre-defined test data (diameters are the most typical metrics used in laboratory experiments to assess tumor size). While we used a specific set of test data for model calibration, this approach can be generalized and applied to different dynamics of organoid development. The method presented here accepts organoids which diameters fit the test data and disregards simulations in which the growth dynamics deviates from the test data. In this way, we can simulate multicellular systems with diverse growth dynamics.

By using an analogy to the structural biology of biomolecules, we provided the morphological classification of multicellular organoids using the radius of gyration (*RGYR)* and accessible surface area (*ASA)*. As a common tool for measuring system’s compactness, *RGYR* applied to organoids informs how dense the tumor mass is and how tightly packed the cells are. The organoid compactness provides a measure of system globularity, such as packing density and intercellular space cavities. Taking this approach, we showed that *RGYR* helps to identify the classes of organoids characterized by different internal cellularity and external branching that may correspond to cell aggressiveness in vivo. The use of the *RGYR* values as a sole classification criterion has raised some concerns because organoids within the same range of *RGYR* attained significantly different morphologies. To overcome this discrepancy, we proposed to also use a concept of the *ASA*, which determines what fraction of the system is exposed to the external microenvironment.

Currently used methods for assessing changes in tumor organoids’ growth rely on measuring their diameters from microscopy images. These measurements, however, may not be accurate, especially if the multicellular structures have irregular shapes. By creating a broad library of organoids morphologies (the *MultiCell-LF* morphochart shown in Fig 3), we proposed to use mathematical modeling for reconstruction of experimental organoid structures and to determine their *ASA* and *RGYR* in a cost- and time-efficient way. The presented computational methods can be combined with measurements obtained from experimental data, i.e., the size of tumor cells can be measured directly, the cell proliferation rates can be determined using cell doubling assays and the organoid’s overall size can be calculated using bright field microscopy images. These values can be mapped on the *MultiCell-LF* morphochart parameter space (Fig 5), which allows for reconstruction of the organoid’s structure in silico and provides the *RGYR* and *ASA* values. This procedure is similar to the acinar morphochart technique we developed previously for determining the functional changes in mutated breast tumor cell lines compared with the parental non-tumorigenic cell line using their 3D morphologies [35, 59–61]. We hypothesized here that currently used treatment protocols for drug dose selection, scheduling and duration could benefit from additional information provided by the *RGYR* and *ASA* values, however further experiments are needed to confirm this postulate.

Tumor tissue irregular architecture is considered as one of the barriers in effective drug penetration though the in vivo tumors [62, 63]. However, similar inefficient penetration has also been observed in tumor organoids and visualized using chemotherapeutic compounds tagged to fluorescent probes [64–66]. We postulate here that *ASA* and *RGYR* may provide prospective metrics for assessing the potential of tumor organoids to be exposed and penetrated by the drug. Some recent studies have discussed the relationship between morphologies of in vivo and ex vivo tumors and their response to drugs [67, 68] showing that there is an interest in developing new measures to assess drug efficacy in tumors and tumor organoids based on their morphology. The organoids considered here (up to 150 μm in radius) are of the size that is below the diffusivity limits of oxygen or nutrients (200–250 μm). However, in larger organoids the gradients of nutrients from spheroid periphery towards its core can develop, that results in the emergence of hypoxic or necrotic regions. As a result, not all cells would have equal access to nutrients and oxygen which may affect their growth, as well as their response to therapeutics.

In this study, we limited the number of intracellular and extracellular heterogeneities, however in the future we are planning to examine how the tumor organoids’ morphology changes when cells have individually-regulated cell cycle, cell-cell interactions and variable sensitivity to contact inhibition. We will also incorporate additional cellular processes, such as cell motility, cell death or secretion of autocrine signals, and evaluate their influence of the emergent organoid’s morphology.

While in the current study we treated the external medium and the interstitial space between the cells as a homogeneous continuum, experimental media and in vivo stroma can contain proteins and fibers of the extracellular matrix that will further impede drug transport. We plan to investigate these factors in the future. Additionally, the morphologies of the tumor organoids can undergo dynamic changes in response to treatments. Not only can they shrink, but they may also become more irregular, if, for example, cell cycle-specific drugs are administered and target only certain cells within the spheroid. Thus, the numerical estimation of drug doses based on the morphological features of tumor organoids (including *ASA* and *RGYR*) could provide a more accurate metric for predicting a tumor’s response to chemotherapy, and for adjusting drug schedules leading to more adaptive and personalized treatment.

## Supporting information

S1 Fig The 3D morphochart of simulated organoids for cells of size 5 microns. The collection of final morphologies simulated for a fixed cell radius *R*_*max*_ = 5 μm, cell division *A*_*div*_ varied between 6 and 17 hours, and cell neighbor number *N*_*neigh*_ between 5 and 22 cells. Three independent simulations were performed for each set of parameters. Only if all three organoids fitted the test data with *R*^2^ >0.9, the one representative oranoid’s morphology is shown. Otherwise, there is an empty space for these parameter combinations.
(TIF)

S2 Fig The 3D morphochart of simulated organoids for cells of size 6 microns. The collection of final morphologies simulated for a fixed cell radius *R*_*max*_ = 6 μm, cell division *A*_*div*_ varied between 9 and 20 hours, and cell neighbor number *N*_*neigh*_ between 5 and 22 cells. Three independent simulations were performed for each set of parameters. Only if all three organoids fitted the test data with *R*^2^ >0.9, the one representative oranoid’s morphology is shown. Otherwise, there is an empty space for these parameter combinations.
(TIF)

S3 Fig The 3D morphochart of simulated organoids for cells of size 7 microns. The collection of final morphologies simulated for a fixed cell radius *R*_*max*_ = 7 μm, cell division *A*_*div*_ varied between 11 and 22 hours, and cell neighbor number *N*_*neigh*_ between 5 and 22 cells. Three independent simulations were performed for each set of parameters. Only if all three organoids fitted the test data with *R*^2^ >0.9, the one representative oranoid’s morphology is shown. Otherwise, there is an empty space for these parameter combinations.
(TIF)

S4 Fig The 3D morphochart of simulated organoids for cells of size 8 microns. The collection of final morphologies simulated for a fixed cell radius *R*_*max*_ = 8 μm, cell division *A*_*div*_ varied between 11 and 25 hours, and cell neighbor number *N*_*neigh*_ between 5 and 22 cells. Three independent simulations were performed for each set of parameters. Only if all three organoids fitted the test data with *R*^2^ >0.9, the one representative oranoid’s morphology is shown. Otherwise, there is an empty space for these parameter combinations.
(TIF)

S5 Fig The 3D morphochart of simulated organoids for cells of size 9 microns. The collection of final morphologies simulated for a fixed cell radius *R*_*max*_ = 9 μm, cell division *A*_*div*_ varied between 16 and 30 hours, and cell neighbor number *N*_*neigh*_ between 6 and 22 cells. Three independent simulations were performed for each set of parameters. Only if all three organoids fitted the test data with *R*^2^ >0.9, the one representative oranoid’s morphology is shown. Otherwise, there is an empty space for these parameter combinations.
(TIF)

S6 Fig The 3D morphochart of simulated organoids for cells of size 10 microns. The collection of final morphologies simulated for a fixed cell radius *R*_*max*_ = 10 μm, cell division *A*_*div*_ varied between 17 and 31 hours, and cell neighbor number *N*_*neigh*_ between 5 and 22 cells. Three independent simulations were performed for each set of parameters. Only if all three organoids fitted the test data with *R*^2^ >0.9, the one representative oranoid’s morphology is shown. Otherwise, there is an empty space for these parameter combinations.
(TIF)
