# Supplementary figures and images for: Morphophenotypic classification of tumor organoids as an indicator of drug exposure and penetration potential
Source: PLoS Comput Biol. 2019 Jul 16;15(7):e1007214. doi: 10.1371/journal.pcbi.1007214 (PMC6660094; doi:10.1371/journal.pcbi.1007214)

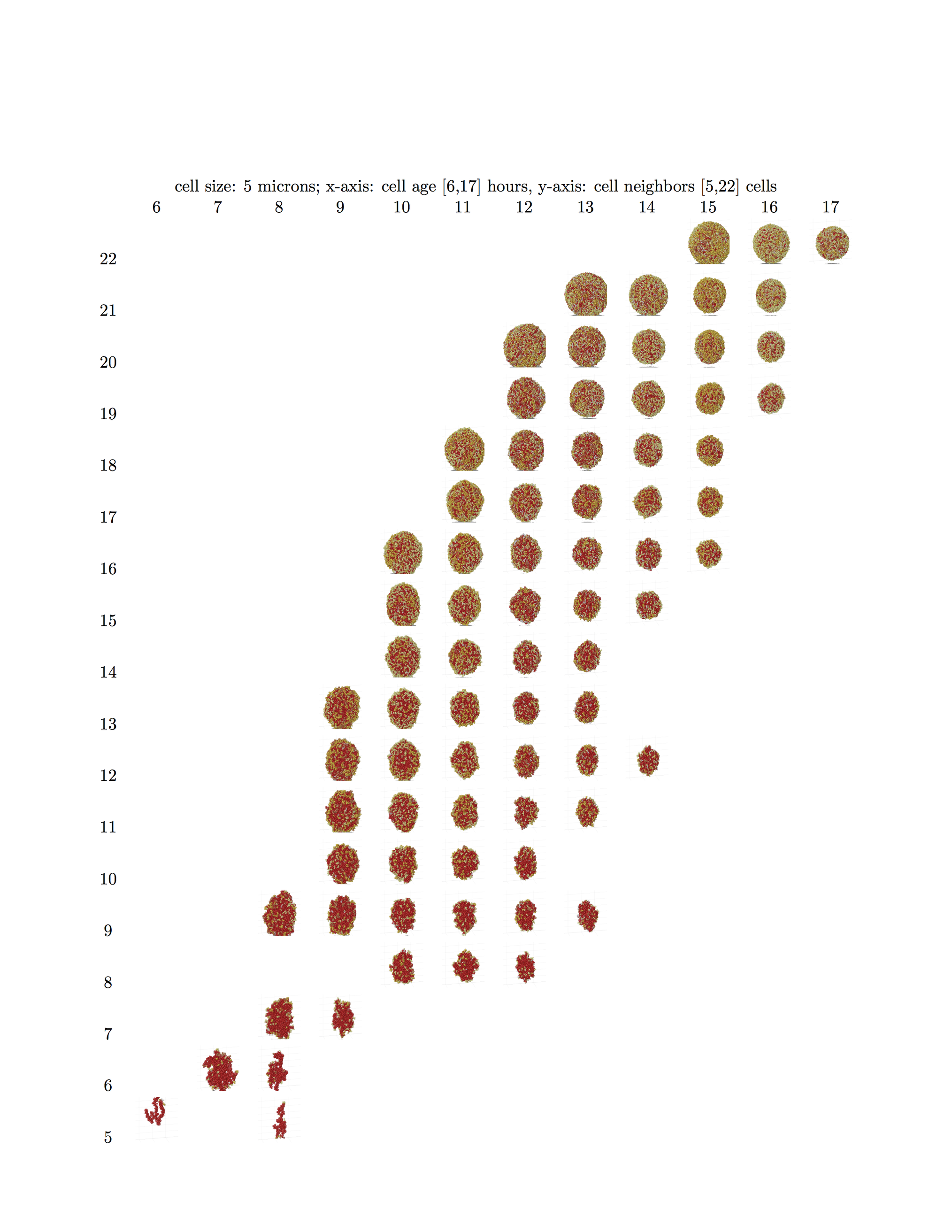

Supplement: S1 Fig — The collection of final morphologies simulated for a fixed cell radius Rmax = 5 μm, cell division Adiv varied between 6 and 17 hours, and cell neighbor number Nneigh between 5 and 22 cells. Three independent simulations were performed for each set of parameters. Only if all three organoids fitted the test data with R2 >0.9, the one representative oranoid’s morphology is shown. Otherwise, there is an empty space for these parameter combinations. (TIF) [file pcbi.1007214.s001.tif]

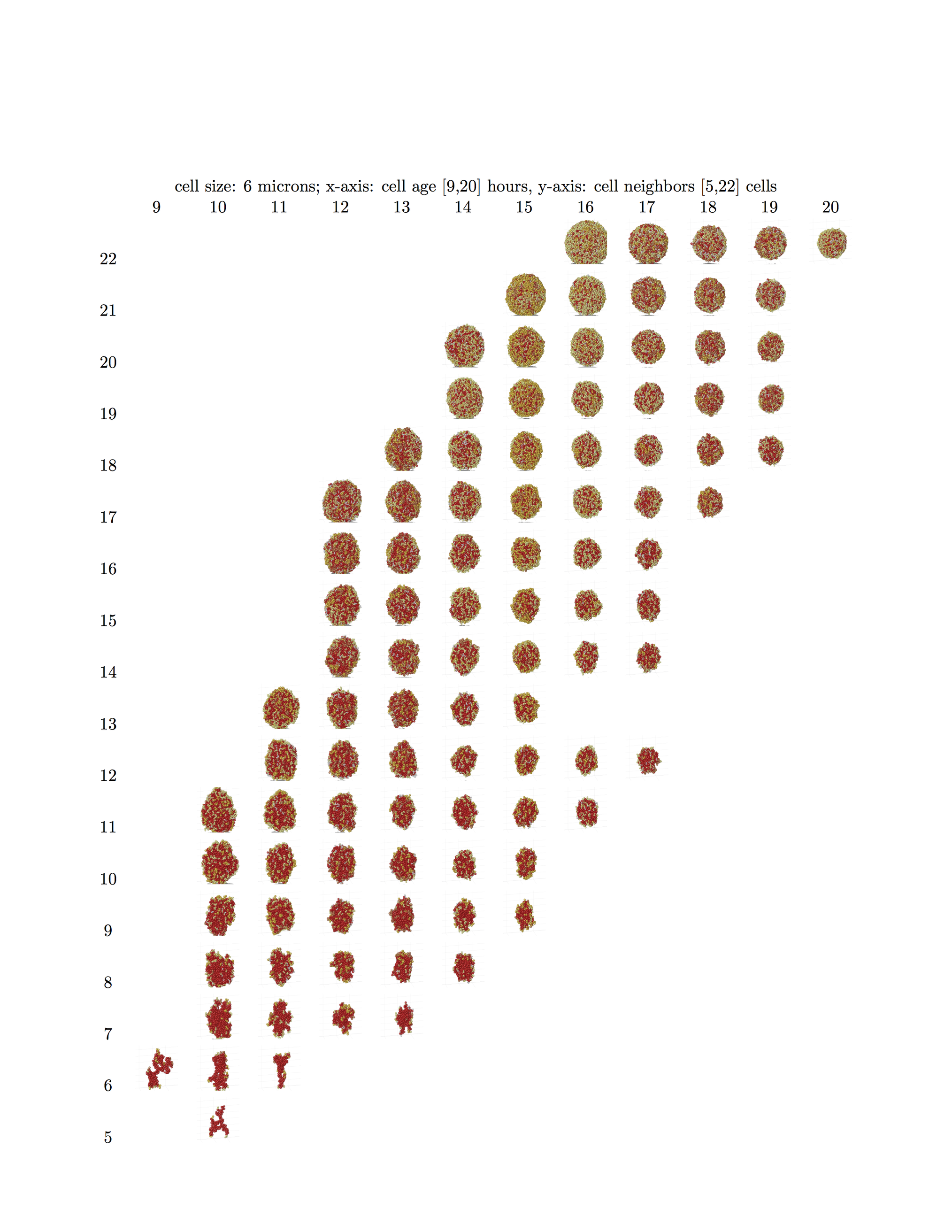

Supplement: S2 Fig — The collection of final morphologies simulated for a fixed cell radius Rmax = 6 μm, cell division Adiv varied between 9 and 20 hours, and cell neighbor number Nneigh between 5 and 22 cells. Three independent simulations were performed for each set of parameters. Only if all three organoids fitted the test data with R2 >0.9, the one representative oranoid’s morphology is shown. Otherwise, there is an empty space for these parameter combinations. (TIF) [file pcbi.1007214.s002.tif]

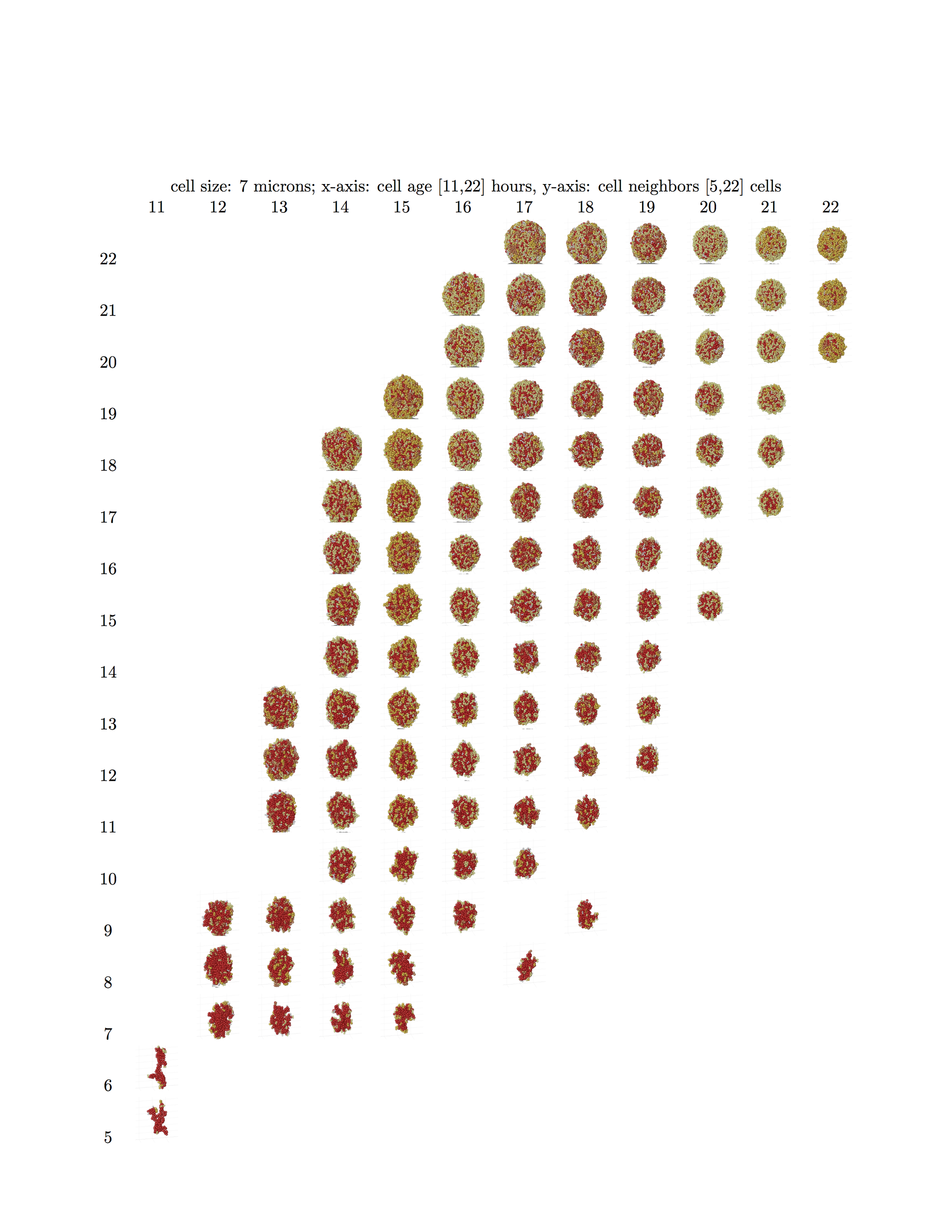

Supplement: S3 Fig — The collection of final morphologies simulated for a fixed cell radius Rmax = 7 μm, cell division Adiv varied between 11 and 22 hours, and cell neighbor number Nneigh between 5 and 22 cells. Three independent simulations were performed for each set of parameters. Only if all three organoids fitted the test data with R2 >0.9, the one representative oranoid’s morphology is shown. Otherwise, there is an empty space for these parameter combinations. (TIF) [file pcbi.1007214.s003.tif]

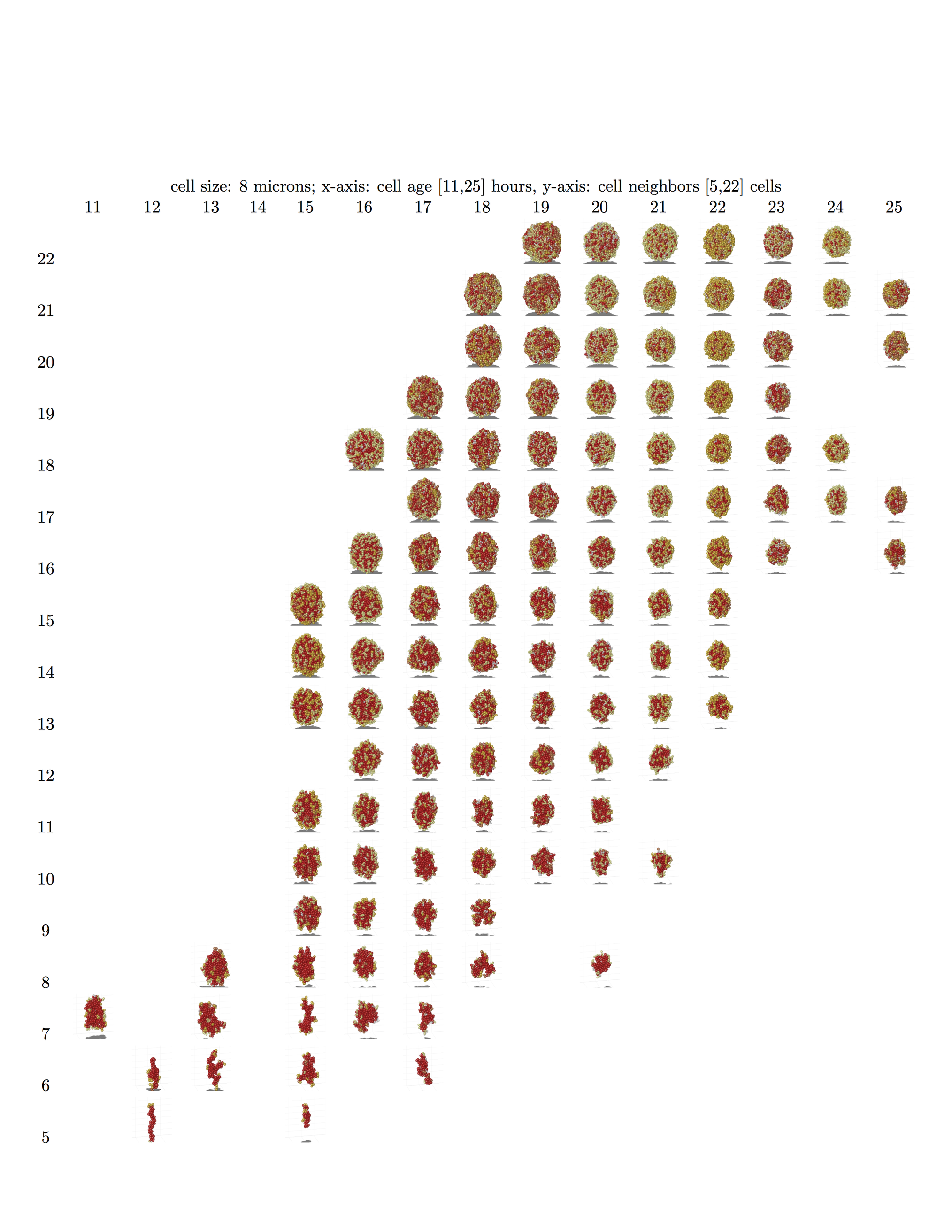

Supplement: S4 Fig — The collection of final morphologies simulated for a fixed cell radius Rmax = 8 μm, cell division Adiv varied between 11 and 25 hours, and cell neighbor number Nneigh between 5 and 22 cells. Three independent simulations were performed for each set of parameters. Only if all three organoids fitted the test data with R2 >0.9, the one representative oranoid’s morphology is shown. Otherwise, there is an empty space for these parameter combinations. (TIF) [file pcbi.1007214.s004.tif]

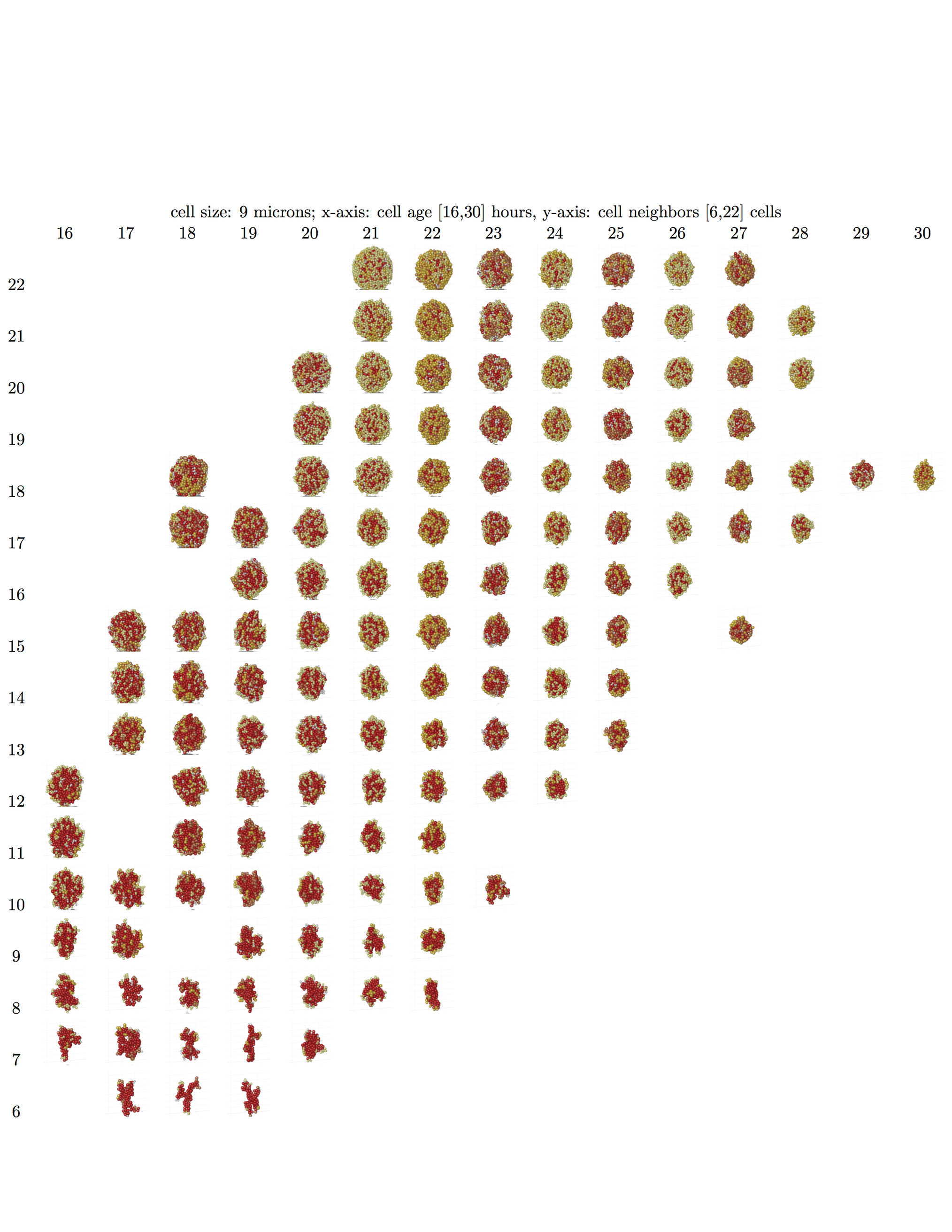

Supplement: S5 Fig — The collection of final morphologies simulated for a fixed cell radius Rmax = 9 μm, cell division Adiv varied between 16 and 30 hours, and cell neighbor number Nneigh between 6 and 22 cells. Three independent simulations were performed for each set of parameters. Only if all three organoids fitted the test data with R2 >0.9, the one representative oranoid’s morphology is shown. Otherwise, there is an empty space for these parameter combinations. (TIF) [file pcbi.1007214.s005.tif]

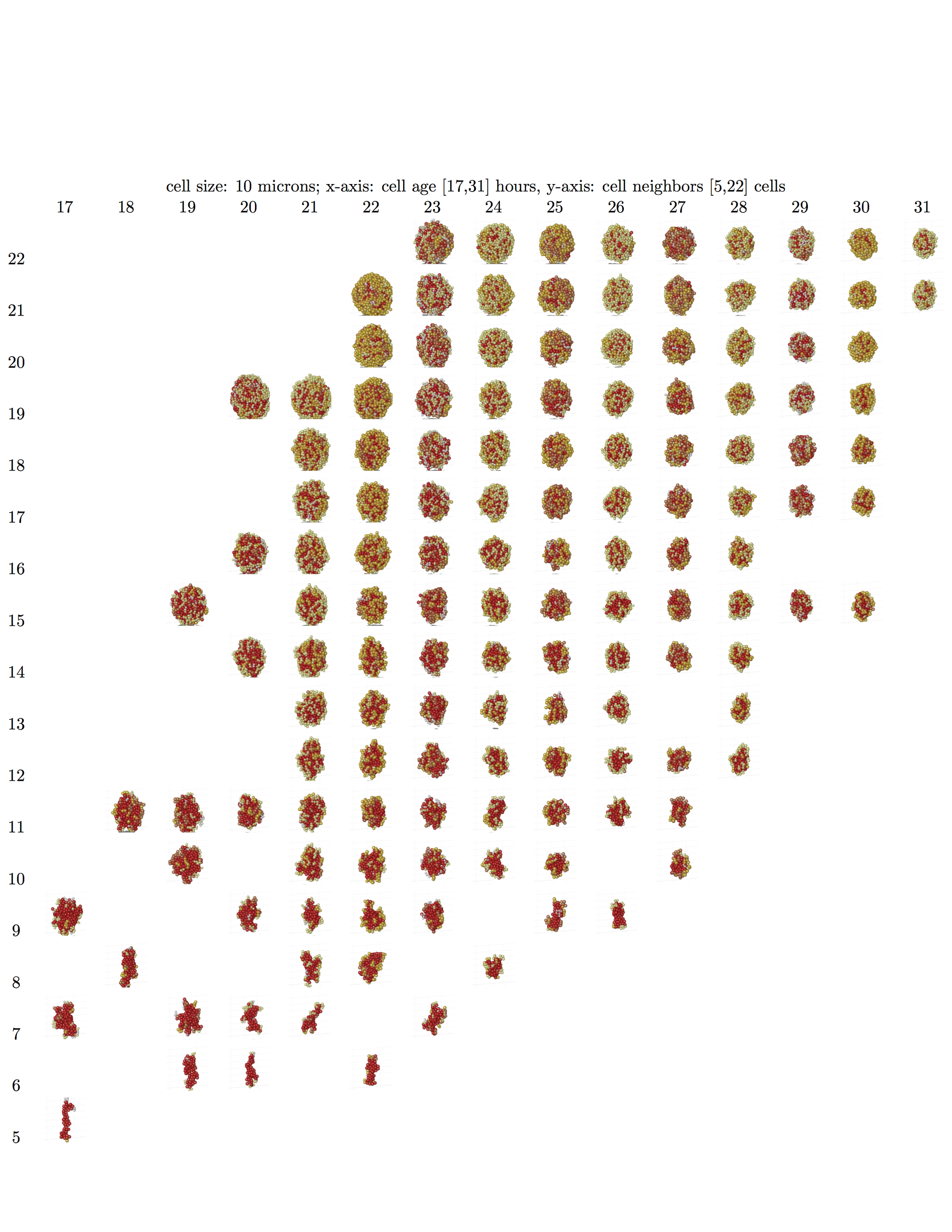

Supplement: S6 Fig — The collection of final morphologies simulated for a fixed cell radius Rmax = 10 μm, cell division Adiv varied between 17 and 31 hours, and cell neighbor number Nneigh between 5 and 22 cells. Three independent simulations were performed for each set of parameters. Only if all three organoids fitted the test data with R2 >0.9, the one representative oranoid’s morphology is shown. Otherwise, there is an empty space for these parameter combinations. (TIF) [file pcbi.1007214.s006.tif]
